# The role of foreign technologies and R&D in innovation processes within catching-up CEE countries

**DOI:** 10.1371/journal.pone.0250307

**Published:** 2021-04-22

**Authors:** Viktor Prokop, Jan Stejskal, Viktorie Klimova, Vladimir Zitek

**Affiliations:** 1 Science and Research Centre, Faculty of Economics and Administration, University of Pardubice, Pardubice, Czech Republic; 2 Institute of Economic Sciences, Faculty of Economics and Administration, University of Pardubice, Pardubice, Czech Republic; 3 Department of Regional Economics and Administration, Faculty of Economics and Administration, Masaryk University, Brno, Czech Republic; Universita degli Studi del Molise, ITALY

## Abstract

Prior research showed that there is a growing consensus among researchers, which point out a key role of external knowledge sources such as external R&D and technologies in enhancing firms´ innovation. However, firms´ from catching-up Central and Eastern European (CEE) countries have already shown in the past that their innovation models differ from those applied, for example, in Western Europe. This study therefore introduces a novel two-staged model combining artificial neural networks and random forests to reveal the importance of internal and external factors influencing firms´ innovation performance in the case of 3,361 firms from six catching-up CEE countries (Czech Republic, Slovakia, Poland, Estonia, Latvia and Lithuania), by using the World Banks´ Enterprise Survey data from 2019. We confirm the hypothesis that innovators in the catching-up CEE countries depend more on internal knowledge sources and, moreover, that participation in the firms groups represents an important factor of firms´ innovation. Surprisingly, we reject the hypothesis that foreign technologies are a crucial source of external knowledge. This study contributes to the theories of open innovation and absorptive capacity in the context of selected CEE countries and provides several practical implications for firms.

## 1 Introduction

Nowadays, innovation is considered a crucial competitive advantage and, thus, an important factor in increasing competitiveness. Innovation leads not only to achieving economic growth and a higher standards of life but also in reaching environmental goals (e.g. [1, 2]). At the same time, hand in hand with the growing importance of innovation, knowledge is becoming one of the main sources of economic growth as it represents the most important factor for creating innovation. The importance of knowledge is emphasized, especially in endogenous growth theories [3–5]. In this context, there is a wide discussion about the transition towards a knowledge economy [6, 7] or the fourth industrial revolution [8]. The process of firm innovation is often referred to as the innovation black-box [9, 10] or the hidden innovation process. Firms´ ability to introduce product innovation is most likely affected by, for example, the availability of in-house and external knowledge, research capacity, type of business ownership, available fixed assets, process innovation, business strategy, training of employees or collaboration with other actors.

Above-described principles are typical primarily for firms innovating in developed western economies. However, firms´ innovating in catching-up Central and Eastern European (CEE) countries have shown in the past that their innovation models differ from those applied in Western Europe. Therefore, we focus in our research on six CEE countries, which (i) have many features in common; (ii) economic performance does not reach the level of the older member states in Western Europe; (iii) joined the European Union in 2004. These are the Czech Republic, Slovakia, Poland, Estonia, Latvia and Lithuania. In addition to being able to face globalization and the changes in society and the economy resulting from the development of modern technologies, as well as other countries in the world, they must also face specific problems and challenges. These challenges are related to the transformation of their economies from centrally planned to market economies in the early 1990s. At this point, we would like to put attention on at least four basic differences between these countries and most other developed states. The first difference is the worse technological equipment of companies. Secondly, the research and innovation system is less developed and does not generate revolutionary research findings and radical innovations. Thirdly, innovation networks are not sufficiently developed, and cooperation within the triple-helix model is not rooted in their environment. Fourth, due to the broken entrepreneurial tradition, people do not have much entrepreneurial skills or spirit and are more risk-averse.

The above-mentioned differences represent a major challenge for current research on the absorptive capacity and open innovation in CEE countries. To the best of our knowledge, prior research focused primarily on the issues of single countries by using Community Innovation Survey (CIS) data. For example, Hajek and Stejskal [11] investigated the influence of R&D cooperation on the creation of spill over effects for sustainable firms in the Czech Republic by using CIS 2008–2010. Prokop et al. [12] used CIS 2010–2012 to analyse effects of innovation cooperation in small CEE countries (Czech Republic, Slovakia, Hungary, Estonia, Slovenia). Kallaste et al. [13] combined CIS 2010 data and telephone interviews to explore open innovation processes in Estonia. In addition, Olaru et al. [14] analysed open innovation practices in Romanian SMEs by using data provided by the Romanian National Statistical Institute of Statistics for the period 2002–2012. Pece et al. [15] used data provided by Eurostat between 2000–2013 to analyse whether the long term economic growth is influenced by the innovation potential of an economy in Poland, Czech Republic and Hungary. However, those studies allow for their interpretation only within the selected spectrum of enterprises in the given countries, which restricts their generalizability. The authors also claim the small sample size as their limitation. This reduces the capability of above studies to fully explore analysed issues. Compared to those and other studies, we use recent data from the World Banks´ Enterprise Survey 2019 that provides unique information about firms´ use of external foreign technologies and, therefore, allows us to expand current scientific knowledge in the case of CEE countries. Additionally, we perform analysis on an aggregated data file including 3,361 firms, which could help increase the ability to generate achieved results.

Moreover, previous research used primarily traditional methods such regression analyses, partial least square structural equation modelling, data envelopment analyses to measure determinants of firms innovation or the efficiency of firms´ during innovation creation. For example, Vásquez-Urriago et al. [16] investigated these effects in Spain, Prokop et al. [17] in the Czech Republic and Slovakia, Niebuhr et al. [18] in Germany, Odei et al. [19] in selected Visegrad countries (the Czech Republic, Hungary and Slovakia) by using regression analyses.

The main motivation of this study is therefore to propose a novel two-staged approach combining artificial neural networks (ANN) and random forests (RF), which overcome previous traditional regression models (see e.g. [20–22]), in terms of accuracy and ability to capture more complex relationships and to handle nonlinear and hierarchical behaviours [23]. It could help us to reveal not the effects of innovation determinants on firms´ innovation performance but the importance of innovation determinants within innovation processes. Moreover, we could answer the question whether firms´ from catching-up CEE countries still rely more on internal sources or whether the importance of external sources has increased and the external absorption capacity of firms has been strengthened.

This research contributes to the theory and practice. From the theoretical perspective, we contribute to the research on the open innovation in the case of firms from Central and Eastern Europe. It was done by including flows of foreign technologies whereas, to the best of our knowledge, there is a lack of similar studies within CEE countries. Second, we contribute to the current research on firms´ internal and external absorptive capacity in CEE countries. This study has also practical contributions. Since most of the previous literature deals with the issue of firms´ internal and external sources of innovation within a single country, the generalizability of their findings can be limited. To overcome this limitation, we include data for 3,361 firms across six CEE countries. This new designation allows us to design practical implications that can be applicable across selected countries.

The remainder of this paper is structured as follows. In the next section, we present a theoretical background for our study. Section 3 provides the research aim, design and methods. Section 4 provides the experimental results. In Section 5, we summarize and discuss the results achieved and propose contributions of this study. In the last part 6, we conclude the paper and limitations and suggestions for future research are presented.

## 2 Knowledge, innovation and cooperation in the era of open innovation

Innovation is generally understood as the creation of something new that has an economic or social significance. We have to distinguish between mere invention and innovation. Innovation differs from invention just in the fact that invention represents some new knowledge (discovery, research finding), while innovation is applied in practice (launched on the market). This is well expressed by the definition of innovation by McCann and Ortega-Argilés [24], who perceive innovation as the process of transforming new ideas into market outputs. The development of innovation theory in recent decades has reformulated the definition and perception of innovation. Innovation is not seen primarily as a process of discovery (i.e., new scientific and technical knowledge), but rather as a non-linear learning process [25–28].

As indicated above, innovation is based on some new knowledge, which may be the result of research and development. The ability to create knowledge and use it in the form of innovation is of the utmost importance for the competitive advantage of companies. The economic theory distinguishes between codified (explicit) and tacit (implicit) knowledge (e.g., [29]). Codified knowledge can be written down or otherwise recorded and thus made available to other people who can acquire or learn it. In contrast, tacit knowledge cannot be recorded and passed on to other people, and one acquires it only through one’s own experience. Tacit knowledge can be acquired through four ways of learning: learning by doing, learning by using, learning by searching and learning by interacting [30].

In particular, tacit knowledge is a source of unique competitive advantage, as it is tied to a specific region and locality and is non-transferable (or difficult and expensive). While codified knowledge can be easily disseminated across a long distance, tacit knowledge is more connected to a given locality (region, state). People’s tacit knowledge, networks of relationships, and experience are becoming increasingly important for the creation of innovations. However, the line between tacit and codified knowledge is not clear. This certainly does not mean that codified knowledge can be absorbed by all who can read. In order to understand some explicit knowledge, people must already have some prior knowledge. In this context, the absorption capacity of the actors is discussed. In other words, much knowledge is only partially codified [29].

Knowledge (especially scientific knowledge) has the character of a public good because it is non-rival and non-excludable [31]. These knowledge features bring externalities that are presented by the knowledge spill overs resulting from R&D [32]. Spill overs generally mean the unintended transfer of knowledge to other innovation players without paying for it. Knowledge spill overs then mean that knowledge created by one actor is used by other actors who do not pay for it either at all or less than the value of this knowledge [33]. Knowledge spillover can occur both between companies themselves [34] and between companies and universities [35]. The fact that knowledge spill overs are not connected with direct compensation brings positive externalities for whole society, but on the other hand, it discourages companies from investing in R&D. It represents the main rationale for public support for R&D [31].

To knowledge spread, there must be some form of proximity among the recipients of knowledge. The importance of different kinds of proximity for the emergence and dissemination of innovation was theoretically developed by Boschma [36, 37]. He distinguishes among geographical, organizational, social, institutional and cognitive proximity (or distance).

The relation between geographical proximity and knowledge transfer is also investigated by Fitjar and Gjelsvik [38]. They pointed out that many companies prefer to work with local universities, as long-distance knowledge transfer is expensive. In addition, local cooperation and frequent face-to-face contact reduce the risk of information loss during transfer. Their research is drawn on the localized knowledge spill overs model, which they extended. Among other things, they argue that knowledge is not only spread from universities to companies but also in other ways. Therefore, cooperation with local universities makes it possible to build regional research capacity that can be used in the future. On the other hand, Laursen et al. [39] point out that the quality of research conducted at a local university plays an important role. They state that being located close to a lower-tier university reduces the propensity for firms to collaborate locally and that co-location with top-tier universities promotes collaboration. According to their research, if companies have a choice, they prefer research quality to geographical proximity. This is especially true for companies using cutting-edge research.

The company’s ability to acquire knowledge from external sources is closely connected with its absorptive capacity. It determines how well the firm is able to acquire and utilize knowledge from external sources [40]. The absorptive capacity is formed by internal and external factors. Prior knowledge and effective organizational routines and communication are considered as vital for absorptive capacity. The company that has some level of knowledge pool is able to understand new knowledge and its usefulness. Absorptive capacity depends on continuous learning through internal R&D or collaboration with external actors (customers, suppliers, competitors, research bodies).

The research and innovation collaboration can bring a significant contribution to increasing companies’ innovation capacity by giving them new ideas and incentives, enabling faster access to resources and enhancing knowledge transfer. At the same time, cooperation enables to share the risks and costs of innovation projects [41]. Thanks to cooperation, companies can share tasks in the innovation process and thus achieve goals that they would not achieve on their own [42]. Innovation collaboration is well explained by the triple helix model, which has been developed by Etzkowitz and Leydesdorff [43, 44]. This model defines three main types of actors influencing innovation (university, industry, government), and analyzes their activities and mutual cooperation. The key idea of this approach is that the creation of innovations is enhanced by the mutual cooperation of the mentioned actors and good knowledge of the needs of the others. Later [45], the model was extended to include cooperation with the civil society (quadruple helix) and the impact of the environment (quintuple helix)

Cooperation on innovation activities is closely linked to the concept of open innovation, the authorship of which is attributed to Chesbrough [46]. The concept of open innovation is based on the idea that not all good ideas will come from inside the organization and not all good ideas created within the organization can be successfully marketed internally. In other words, external ideas and external paths to market have the same importance as internal ideas paths to market in the framework of the closed innovation. Later, the definition was supplemented by the intentionality of the knowledge flows [47] and pecuniary and non-pecuniary knowledge flow mechanisms [48, 49].

There are two basic modes of open innovation [47]. Inbound open innovation focuses on the process of innovation emergence and emphasizes that companies do not have to rely solely on their own research and the creation of new knowledge, but can use the knowledge that has arisen elsewhere. In contrast, outbound open innovation focuses on the process of spreading innovation and distributing innovative products to customers. This mode highlights that companies do not have to rely solely on their own paths to the market, but that they can work with external organizations that have better business models to commercialize new technologies.

Chesbrough and Crowther [47] have shown that the concept of open innovation is not only applicable in high-tech industries but is also used in traditional and less knowledge-intensive industries. The same authors also point out that the company does not have to entrust all research activities to external organizations. Innovative companies usually carry out their own research and, at the same time, use external research collaboration. This is also related to the absorption capacity of companies that was already discussed above.

## 3 Research aim, design and methods

### 3.1 Research aim and hypotheses

The aim of this study is to propose a novel two-staged artificial neural networks-random forests approach that allows us to identify the factors that are vital for firms´ innovation creation within countries from the Central and Eastern Europe. These countries are often seen as lagging behind the countries from the Western Europe, for example because of their worse technological equipment of firms, less developed innovation networks and social capital, lower level of trust among economic entities and mental lock-in.

Following above arguments, we define two research hypotheses. First, we focus on the importance of internal and external knowledge sources. We expect that internal sources are crucial in stimulating firm’s innovation capabilities and absorptive capacity, help to better understand innovation process within a firm and enhance innovative outputs and competitive advantage, specifically within CEE countries, which often lack of funds and insufficient incentives to cooperate [50–52]. Therefore, we hypothesize that:

*H*_*1*_: *Innovators in the catching-up CEE countries depend more on internal knowledge than on external knowledge*.

Second, there is a growing question about the importance of external (foreign) technologies. Despite we expect that internal knowledge sources are more important than external ones for firms within selected CEE countries, we still expect significant role of foreign technologies within innovation processes. We found support for this argument for example in the work of Hu et al. [53], which examined the relationship between internal R&D investments (as a proxy for absorptive capacity) and positive effects of foreign technology, using data on 35 Chinese industrial sectors from 2001 to 2010. Moreover, Parisi et al. [54] showed that firms´ R&D is a crucial factor in facilitating the absorption of new technologies in the cases of Italian manufacturing firms. In addition, Sharma et al. [55] stated that there could be a complementary relationship between firms´ R&D efforts and foreign technology.

Following above arguments and taking into account that innovation is costly, risky, and path-dependent, we can expect, according to Fu et al. [56], that it is more efficient for lagging countries to acquire foreign technology created in more developed countries. The authors state that if innovations are easy to spread and absorb, a technologically lagging country could catch-up faster by absorbing the most advanced foreign technologies. Therefore, we hypothesize that:

*H*_*2*_: *Foreign technologies represent a crucial source of external knowledge for innovators in catching-up CEE countries*.

### 3.2 Data description

For the purpose of this study, we use the latest available data from the Enterprise Survey (ES) 2019, provided by the World Bank, which includes various topics focused on business environment (access to finance, corruption, infrastructure, crime, competition, and performance measures). ES provides data about enterprises in the manufacturing and service sectors in every country of the world by using a global methodology that includes standardized survey instruments and a uniform sampling methodology (to see more www.enterprisesurveys.org/en/methodology). We focus on firms in selected Central and Eastern Europe (CEE) countries, concretely within Central Europe countries—the Czech Republic, Slovakia, and Poland and within Baltic States—Estonia, Latvia, and Lithuania. In total, we analyze 3,361 firms from these countries. More details about them are stated in Table 1.

**Table 1:** Characteristics of analyzed firms in the sample.

Country	Number of cases	Innovative	Using external knowledge	Using Internal R&D expenditures	Using Foreign Technologies
Czech Republic	502	39.4%	15.3%	36.9%	16.1%
Slovakia	429	16.6%	9.1%	12.8%	33.1%
Poland	1,353	20.5%	7.7%	8.3%	12.9%
Estonia	360	40.3%	22.5%	27.2%	11.4%
Latvia	359	49.1%	22.6%	21.7%	26.5%
Lithuania	358	32.7%	25.4%	7.8%	12.8%
**Total**	**3,361**				

The above-mentioned methodological procedures are explained in more detail in the following text. Dependent and independent variables are shown in Table 2.

**Table 2:** Variables of the proposed model.

Dependent variable (Throughput)	Description (question from the Enterprise Survey 2019)
Firms´ innovation (INN)	During the last three years, has this establishment introduced new or improved products or services?
**Independent variables–Internal sources**	
New pro-innovative processes (PROC)	During the last three years, has this establishment introduced any new or improved process? These include methods of manufacturing products or offering services; logistics, delivery, or distribution methods for inputs, products, or services; or supporting activities for processes?
Training (TRAIN)	Over fiscal year, did this establishment have formal training programs for its permanent, full-time employees?
Internal R&D exp. (RRDIN)	Over the last three years, did this establishment spend on research and development activities within the establishment?
Strategy (STR)	Does this firm have formalized, written business strategy with clear key performance indicators?
**Independent variables–External sources**	
External knowledge (ROEK)	Over the last three years, did this establishment spend on the acquisition of external knowledge? This includes the purchase or licensing of patents and non-patented inventions, know-how, and other types of knowledge from other businesses or organizations.
External R&D exp. (RRDEX)	Over the last three years, did this establishment spend on research and development activities contracted with other companies?
Foreign technologies (TECH)	Does this establishment at present use technology licensed from a foreign-owned company, excluding office software? Acquisition of external (foreign) technologies could help firms to reduce the costs and uncertainties related to expensive and risky innovations [57].
Ext. intangible assets (INTAS)	In fiscal year, did this establishment purchase or acquire any trademarks, copyrights, patents, licenses, service contracts, franchise agreements, or other intangible assets?
Ext. fixed assets (FIXAS)	In fiscal year, did this establishment purchase any new or used fixed assets, such as machinery, vehicles, equipment, land or buildings, including expansion and renovations of existing structures?
**Independent variables–Controls**	
Domestic ownership (DOMOWN)	Domestic ownership including private domestic individuals, companies or organizations
Domestic markets (MARK)	Did this establishment sold its main product at domestic market?
Enterprise group (ENTGP)	Is this firm part of a business membership organization, trade association, guild, chamber of commerce, or other business support group?
Industry (MANUF)	Did this establishment sold its main product in manufacturing sector?

Source: authors’ own elaboration based on ES questionnaire.

### 3.3 A two-staged model proposal

**Fig 1:**
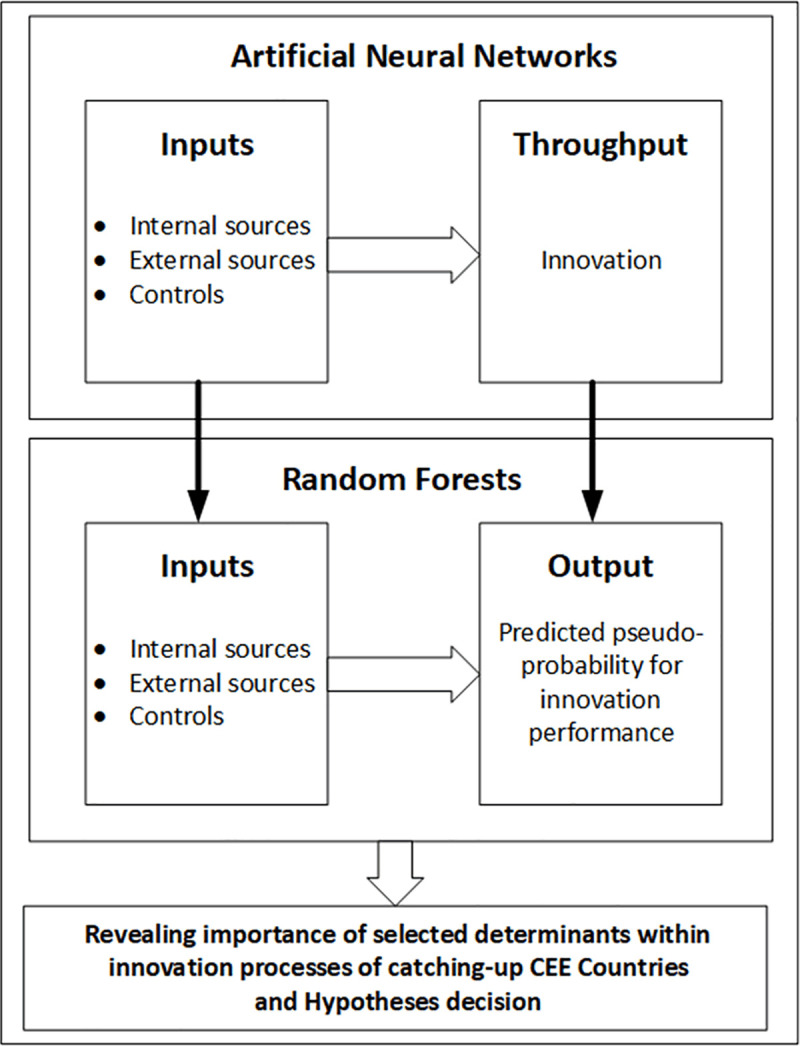
Proposed two-staged ANN-RF model.

A proposed research process consists of two stages, as shown in Fig 1. First, by using the World Bank Enterprise Survey data and ANN approach, we model firms´ innovation performance (firms´ innovation represent throughput) and predict its pseudo-probability. Second, by using the same input data, we perform Random Forests approach to reveal the importance of selected variables in the process of innovation creation (represented by predicted pseudo-probability for firms´ innovation performance).

#### A) Artificial neural networks

Artificial neural networks are part of the so-called artificial intelligence and represent nonlinear mathematical models that are able to simulate arbitrarily complex nonlinear processes that relate inputs and outputs of any system and which can be associated with a network of neurons organized in layers [58, 59]. More specifically, according to Dahikar and Rode [60], ANN is defined by the interconnection pattern between different layers of neurons, learning process for updating the weights of the interconnections and activation function that converts a neuron’s weighted input to its output activation.

According to Tu [61], neural networks (NN) have the same goals as traditional logistic regression models in predicting an outcome based on the values of predictor variables. There are also some differences between them. In comparison for example with regression models, neural networks have the ability to learn mathematical relationships between a series of inputs and outputs. ANN consists of L+1 layers where 0 is the input layer of source nodes (inputs), output layer is layer L, the layers 0<l<L are one or more hidden layers of computation nodes/neurons [62, 63]. The number of neurons in the 0—input and the L—output layer depends on the number of input and output variables.

Generally, the input vector *x* = (*x*_1_,*x*_2_,…,*x_N_*)^*T*^ is transformed to an intermediate vector of hidden variables *l*, using the activation function *φ*_1_. The output *l_j_* of the *j*th node in the hidden layer is obtained as follows [64]:

$$l_{j}=\varphi_{1}(\sum_{i=1}^{N}w_{i,j}^{1}x_{i}+b_{j}^{1})$$ (1)

where ${b_{j}^{1}}_{}$ and ${w_{i,j}^{1}}_{}$ represent the bias and the weight of the connection between the *j*th node (in the hidden layer) and the *i*th (input) node and the superscript 1 represents the first connection between the input and hidden layers.

The output vector *y* = (*y*_1_,*y*_2_,…,*y_Q_*)^*T*^ is obtained from the vector of intermediate variables *l* through a similar transformation using an activation function *φ*_2_ at the output layer.

For example, the output of the neuron *k* can be expressed as follows:

$$l_{k}=\varphi_{2}(\sum_{l=1}^{M}w_{l,k}^{2}x_{i}+b_{k}^{2})$$ (2)

where the superscript 2 represents the second connection between the neurons of the hidden and the output layers.

**Fig 2:**
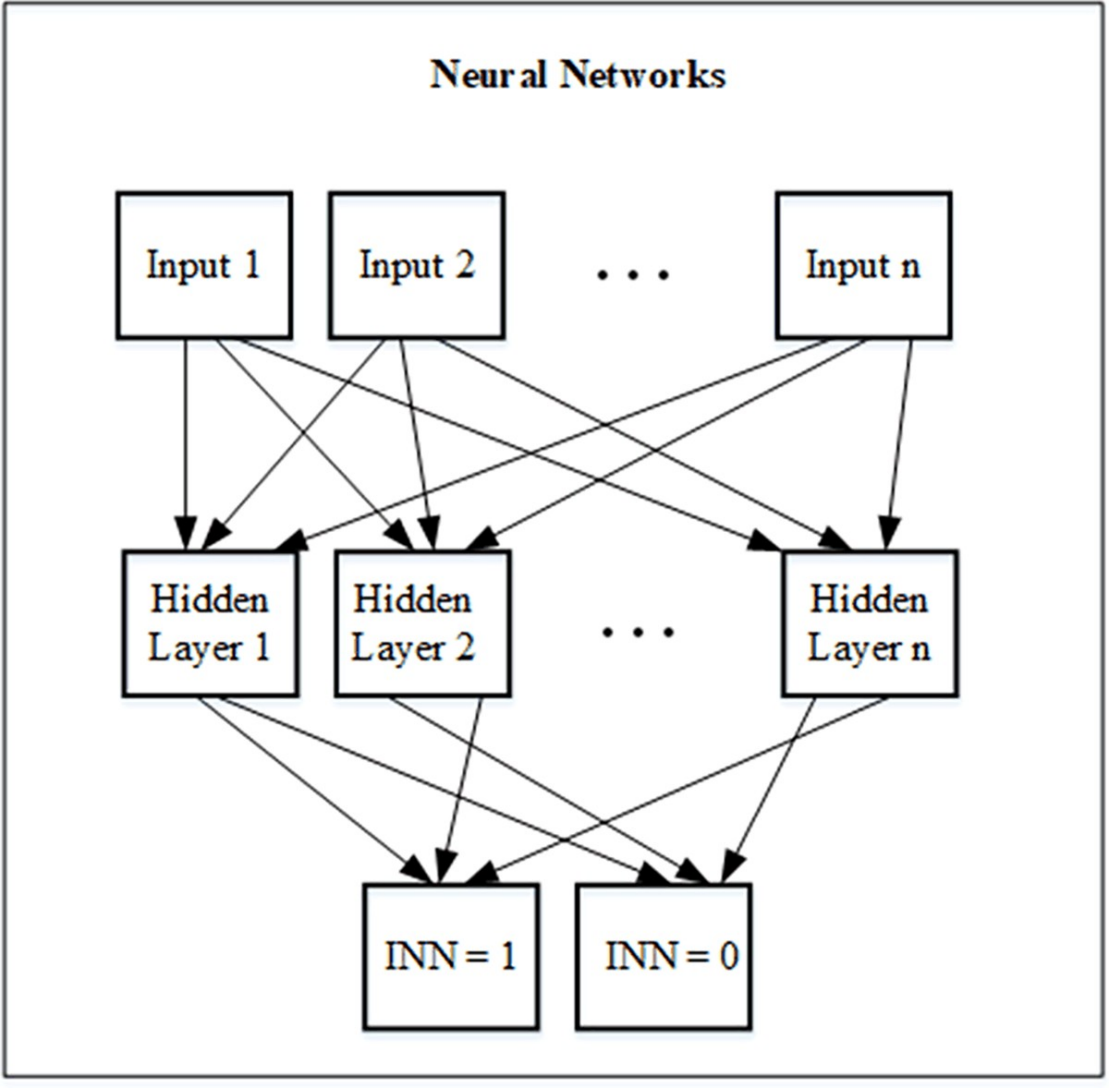
Proposed neural networks model.

In this article, we use NN architecture, which is a multi-layer perceptron (MLP) that has been commonly used for modelling, prediction, classification, or control in many engineering applications [65, 66]. Fig 2 shows proposed Neural Networks model operating with Hidden Layers of innovation processes. The input layer of proposed NN consists of inputs presented in Table 2, the output layer is seen as the classified result that infers products or services innovation or non-innovation, and the hidden layer is the classifying layer in order to transform inputs to outputs.

ANN are robust to handle noise, outliers and overfitting and have several advantages such as the ability to (i) handle large datasets; (ii) approximate non-linear relationships; (iii) generalize from relatively imprecise input data [67].

To identify important factors influencing firms introducing of innovation (INN = 1), we follow knowledge about the predicted pseudo-probability for firms´ innovation performance obtained from ANN and suggestions of Frosst and Hinton [68] and use random forests that has proven to be a highly accurate classifier and that represent an ensemble classification algorithm which consists of a collection of decision trees [69] that, in some cases, also outperformed ANN with a lower error rate and higher accuracy rate (see [70]).

#### B) Random forests

In the second stage, we use predicted pseudo-probability for firms´ innovation performance prediction provided by ANN and random forests (RF), which were developed by Breiman [71]. Random forests are the improvement of the bagging principle by suppressing the correlation among of fitted trees to form an ensemble of classification and regression tree (CART)-like classifiers [72]. This is achieved by forcing each split to utilize only a small subset of predictors. In general, the basic idea of bagging principle is similar to combine the outputs of many weak (base) learners to produce a powerful overall final result (majority voting). The predictive power of these individual learners is weak and prone to overfitting but combining many such weak models in an ensemble lead to an overall much improved result. In addition, the bagging principle, introduced also by Breiman [73], is based on fitting of many large classification trees to bootstrap-resampled versions of the training data (samples with repetition) and classify by majority voting.

According to Breiman [74] and Gislason et al. [72], RF is a general term for ensemble methods using tree-type classifiers:

$$\left\{h(x,\Theta_{k}),k=1,\ldots ,\right\}$$ (3)

where {Θ_k_} are independent identically distributed random vectors and *x* is an input pattern.

*In training, the Random Forest algorithm creates multiple CART-like trees [75], each is trained on a bootstrapped sample of the original training data, and searches only across a randomly selected subset of the input variables to determine a split (for each node). For classification, each tree in the Random Forest casts a unit vote for the most popular class at input x. The output of the classifier is determined by a majority vote of the trees* [72].

Classification trees are understood as an efficient classification tool [67]. It repeatedly splits the data set according to a criterion that maximizes the separation of the data, resulting in a tree-like structure [76]. Generally, decision trees are graphical representations of a procedure for classifying or evaluating the alternatives of interest and clearly show how to reach a decision [77]. The main advantage of decision trees is that they are not black-box models [78], so they can be easily expressed as rules. Moreover, decision trees are a commonly used model to help in discovering, understanding, and communicating the structure of such decision problems [79].

## 4 Experimental results

This chapter is divided into two parts. First, we present an original method for modeling firms´ innovation performance by using artificial neural networks. Second, we provide results of random forests that indicate importance of innovation determinants within firms’ innovation processes in catching-up CEE countries.

### 4.1 Artificial neural networks model for CEE countries

To model innovation performance within CEE countries, we applied the most commonly used perceptron neural network that consists of input layer, hidden layer and output layer. For the input and hidden layers, multilayer perceptrons and sigmoid activation functions were used [80]. According to Leong et al. [81], several rounds of the learning process can lead to the decrease of the errors, and the accuracy of the prediction can be further improved. Therefore, we subsequently performed two models to compare the results of neural networks operating with different number of hidden layers. First model consists of one hidden layer (Model 1a), whereas we chose a random distribution of input data, where 69.5% of the data were employed as training data and 30.5% as test data (case processing summary is shown in Table 3). Second model consists of two hidden layers (Model 2a). We again chose a random distribution of input data, where 69.7% of the data were employed as training data and 30.3% as test data (case processing summary is shown in Table 4).

**Table 3:** ANN case processing summary–Model 1a.

	Number of cases	Total percentage	Correct predictions
Training sample	2,335	69.5%	73.7%
Testing sample	1,026	30.5%	73.1%
Valid	3,361	100.0%	
Excluded	0		
Total	3,361		

**Table 4:** ANN case processing summary–Model 2a.

	Number of cases	Total percentage	Correct predictions
Training sample	2,341	69.7%	74.1%
Testing sample	1,020	30.3%	72.6%
Valid	3,361	100.0%	
Excluded	0		
Total	3,361		

The structure of the neural network was created using the SPSS program while we set up automatic selection of the architecture of the number of neurons in the hidden layer and online approach to form the learning algorithm of NN. Model 1a reached overall correct predictions percentage of training data was 73.7%, and 73.1% of testing data. Model 2a reached overall correct predictions percentage of training data was 74.1%, and 72.6% of testing data.

**Fig 3:**
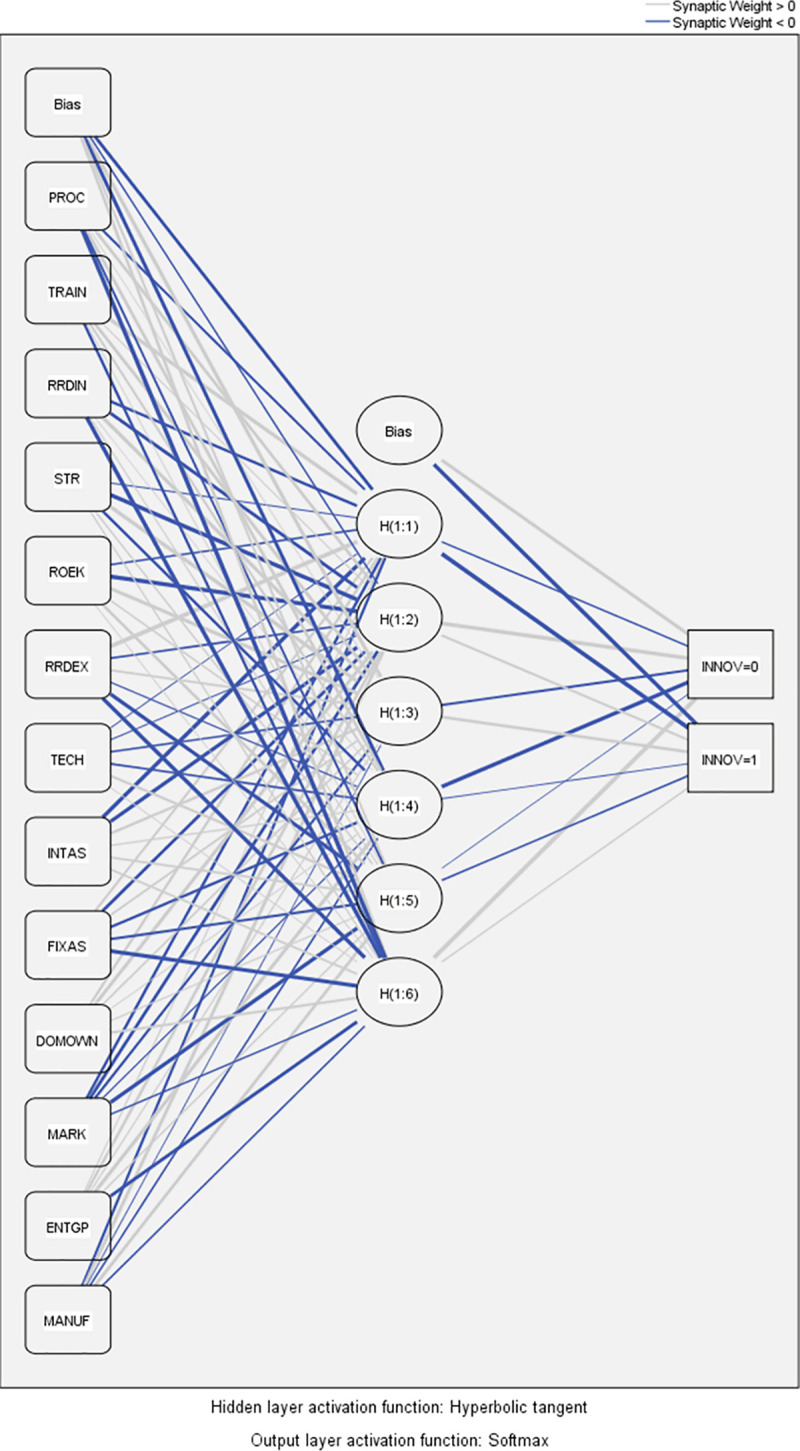
Artificial neural network diagram–Model 1a.

**Fig 4:**
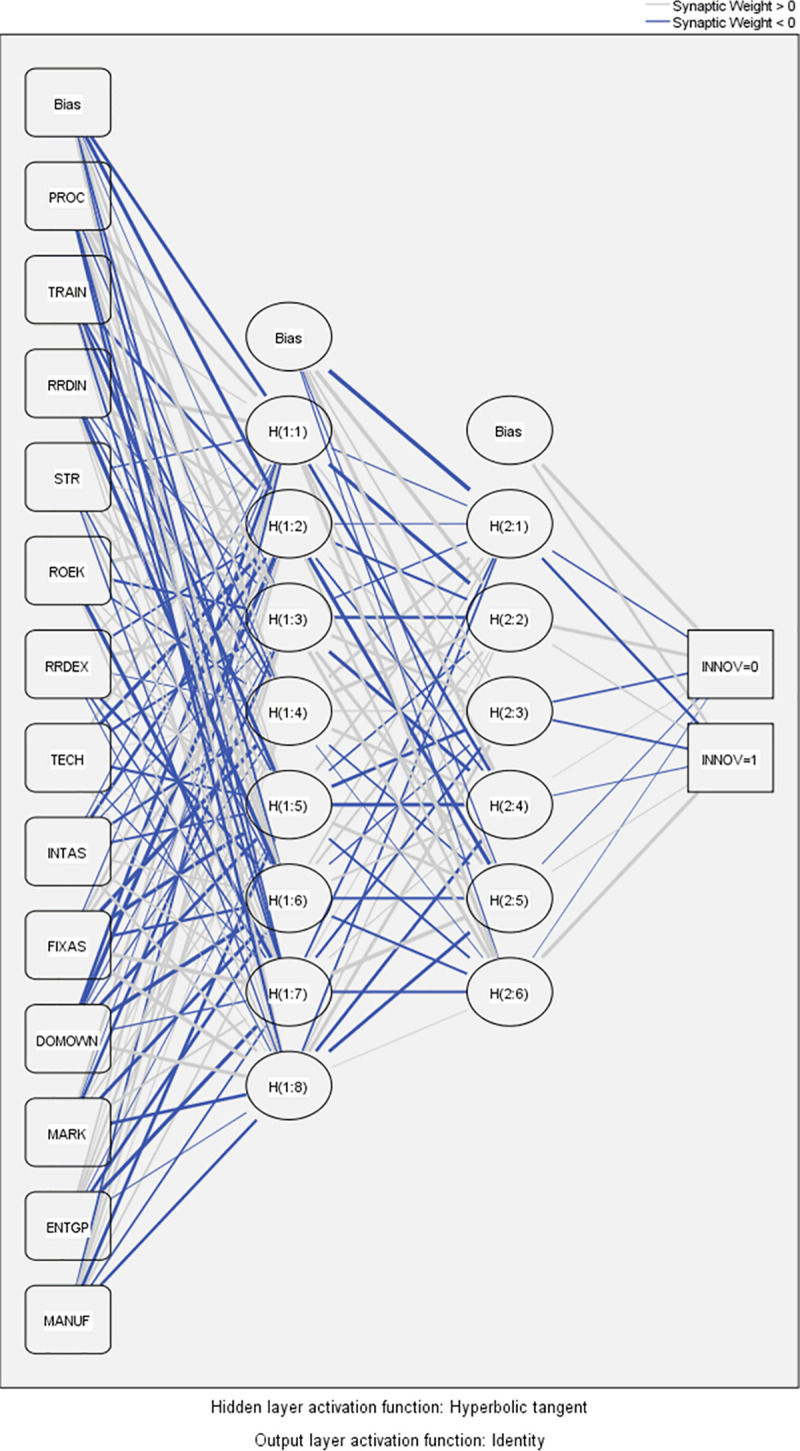
Artificial neural network diagram–Model 2a.

Figs 3 and 4 show performed ANN models.

Following suggested procedure shown on Fig 1, we used predicted pseudo-probabilities for firms´ innovation and employed the random forests approach. We used the same input variables as in the previous parts and the results of ANNs. It allowed us to reveal significant factors influencing firms’ innovation outputs and to decide on defined hypotheses.

### 4.2 Random forests based on the ANN results for CEE countries

In this stage, we used classification random forests based on the results of ANN Model 1a (RF Model 1b) and ANN Model 2a (RF Model 2b). The number of trees for both presented models were 500. Number of variables tried at each split were 3. Each tree grown to the largest extent possible and there was no pruning of the trees. OOB (out-of-bag) estimate of error rate for Model 1b were 3.42%, and 2.56% for Model 2b. Tables 5 and 6 show confusion matrixes for both models.

**Table 5:** RF confusion matrix–Model 1b.

		Observed		
		0	1	class. error
Predicted	0	2873	31	0.01067493
	1	84	373	0.18380744

**Table 6:** RF confusion matrix–Model 2b.

		Observed		
		0	1	class. error
Predicted	0	2752	25	0.009002521
	1	61	523	0.104452055

**Fig 5:**
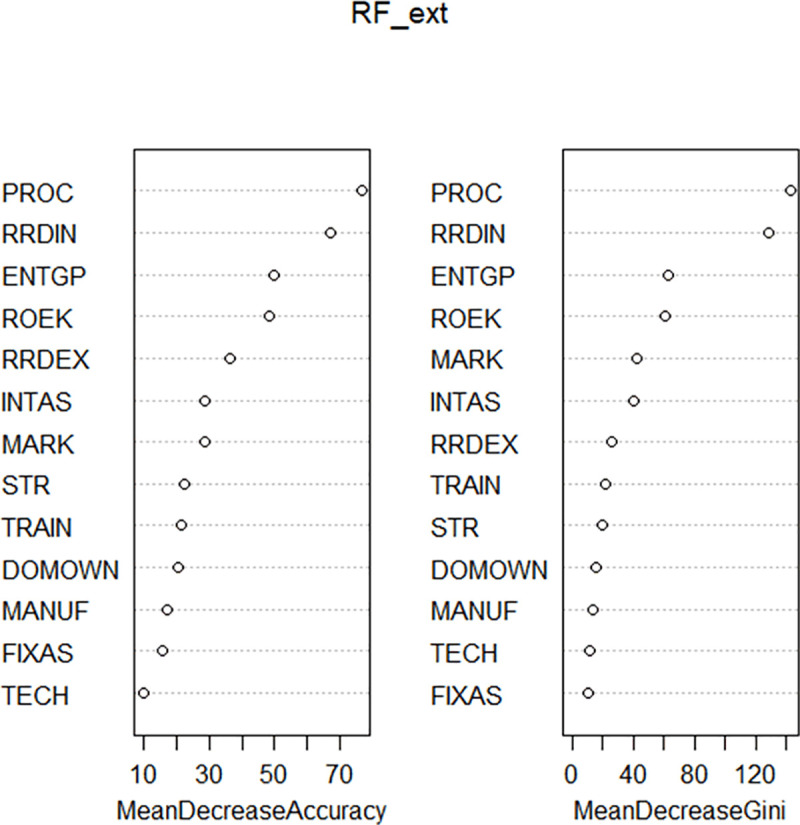
Importance of selected innovation determinants–Model 1b.

**Fig 6:**
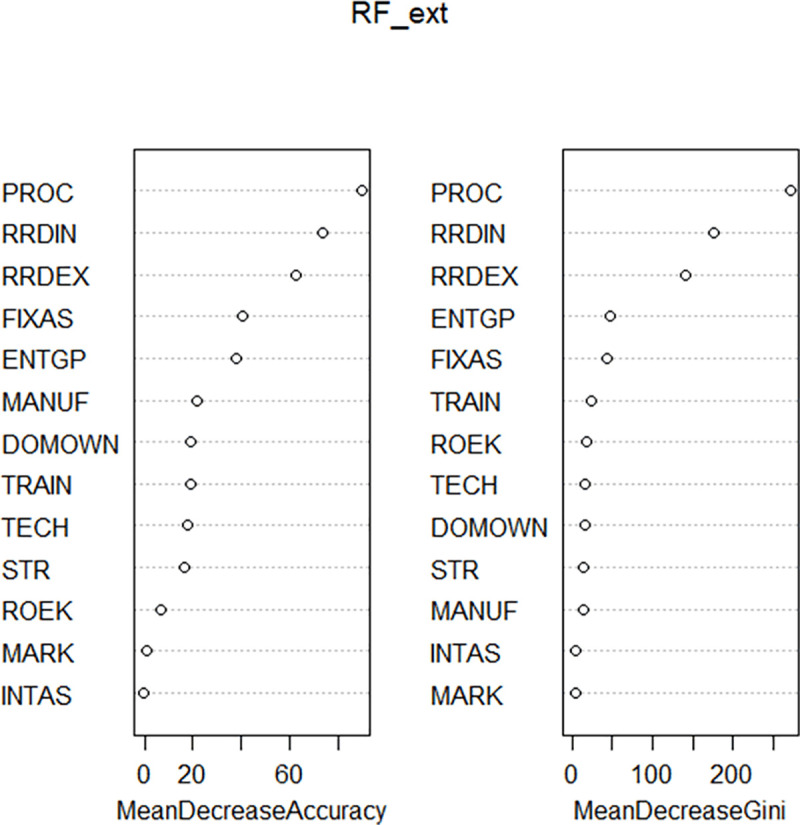
Importance of selected innovation determinants–Model 2b.

Figs 5 and 6 shows importance of selected innovation determinants within CEE countries.

The above results clearly indicate the importance of firms´ internal pro-innovative processes including methods of manufacturing products or offering services; logistics, delivery, or distribution methods for inputs, products, or services; or supporting activities for processes. Moreover, we show that internal research and development activities represent more important factor than external research and development or acquisition of external knowledge including the purchase or licensing of patents and non-patented inventions, know-how, and other types of knowledge from other businesses or organizations for firms´ innovation.

The results also show that the use of external foreign technologies is at a low level of importance for innovators from catching-up CEE countries.

Following above findings, on the one hand, we can **accept the hypothesis *H***_***1***_ that innovators in the catching-up CEE countries depend more on internal knowledge than on external knowledge. On the other hand, we **reject the hypothesis *H***_***2***_ that foreign technologies represent a crucial source of external knowledge for innovators in catching-up CEE countries.

## 5 Discussion and contributions

### 5.1 Discussion of the results

In this study, we have designed a new way of analysing firms´ innovation performance using a two-staged artificial neural networks-random forests approach. On the one hand, ANNs reach the most accurate results in comparison with other techniques including for example logistic regression, radial basis function, self-organizing feature maps [82] and whose learning capability makes them very suitable for different specific applications including pattern recognition or data classification [83]. On the other hand, RF represent a combination of tree predictors that is robust and rarely overfits and, therefore, it hence yields highly accurate predictions. Moreover, RF can handle nonlinear and hierarchical behaviours [23].

We show that firms´ pro-innovative processes and internal R&D are more important than external R&D and knowledge sources in the case of firms´ from catching-up CEE countries. In addition, participation in the firm group was revealed as other important factor. It can therefore be assumed that firms are capable and willing to absorb certain external knowledge, but only from long-term partners, such as from the same firm group. These results are consistent with the findings of Belso-Martínez et al. [84], which pointed out the significance of group structures in the case of Spanish clusters. The authors state that group structures could lead to better innovation practices because knowledge pooled by the group members through their networks. It can subsequently enhance firms´ creativity and innovation processes. These results are also consistent with studies focused on the case of selected CEE countries. Odei and Stejskal [85], on the one hand, proved that Czech and Hungarian firms’ collaborations with partners from enterprises groups have a positive influence on product innovations. On the other hand, they showed that collaborations with R&D partners for process innovations produced mixed results for both countries.

Surprisingly, we did not prove the importance of foreign technologies for firms´ innovation. These results are in contrast with above mentioned study of Hu et al. [86] that confirmed significant relationship between firms´ internal R&D investments and foreign technology. Moreover, these results are inconsistent with previous findings of Parisi et al. [54], which pointed out that firms´ R&D facilitate the absorption of new technologies. Moreover, we did not support the results of Park and Choi [86], which stated that firms’ participation in enterprise group enables firms to easily obtain and learn their various know-how and technologies. The results of our study show that firms in CEE countries still face the challenge of absorbing knowledge and technology from external partners that is crucial because wrong firms´ technology-acquisition innovation strategy, based, e.g., on industrial machinery and equipment acquisition, could lead to the creation of negative effects on innovation outputs [87], e. g. crowding-out effect (in principle, the same negative effect as the innovation paradox [88]).

On the other hand, above results are consistent with Fu et al. [56], which showed that the benefits of foreign technology diffusion can only be delivered, if there is a presence of modern institutional and governance structures and conducive innovation systems in the domestic country. The authors proved these statements in the case of emerging economies. Moreover, Sharma [80] showed, in the case of manufacturing firms from Bangladesh, that there could be a substitutive relationship between firms internal R&D and foreign technology transfer. The authors also showed that firms which engaged in both activities, R&D and foreign technology transfer, have a lesser labour productivity.

### 5.2 Theoretical and practical contributions

This study contributes to the theory in two ways. First, according to Chesbrough [89], this study contributed to expanding the current theoretical state of knowledge in the field of open innovation, which demands new external ideas and technologies to advance firms´ technology, absorptive capacity and productivity in the case of firms from Central and Eastern Europe. It was done primarily by including foreign flows of technologies whereas, to the best of our knowledge, there is a lack of similar studies within CEE countries. The results of our study clearly indicate that firms´ are still locked-in and that these firms´ do not fully profit from inbound open innovation. It results in the fact that firms have to rely on their own research and the creation of new knowledge and cannot use the knowledge that has arisen elsewhere.

Second, we contribute to the theory of absorptive capacity, which determines, according to [40], how well the firm is able to acquire and utilize external knowledge. While the absorptive capacity is formed by internal and external factors and depends on continuous learning through internal R&D or collaboration with external actors, we clearly proved that firms´ internal absorptive capacity plays a key role within innovation processes in selected catching-up CEE countries. According to Lewin et al. [90], internal absorptive capacity capabilities refer to managing the processes of internal variation, selection, and replication, described in evolutionary economics. On the other hand, external absorptive capacity capabilities include the management of exploration for new knowledge in the external environment and its assimilation.

The results of this study also provide several interesting practical implications, specifically for firms from CEE countries. First, since most of the previous literature deals with the issue of firms´ capability to absorb and use external and internal resources to innovation within a single country, the generalizability of their findings can be limited. To overcome this limitation, we included data for 3,361 firms across six CEE countries. This new designation allows us to design practical implications that can be applicable across selected countries.

As we showed above, external technologies do not represent important source for firms´ innovation in selected CEE countries. To increase the possibility for firms to use external (foreign) technologies and thus benefit from new knowledge and new sources of competitive advantage, there is a need to support firms´ internal and external absorptive capacity and employees’ abilities. It could be done, for example, by formal training programs and by building internal social capital that is recognized as crucial for firms´ innovation activities because it promotes mutual trust, interaction, cooperation, and information sharing [91]. Following the arguments of Fu et al. [56], to support the benefits of foreign technology diffusion, there is a need to build a modern institutional and governance structures and conducive innovation systems in catching-up CEE countries.

Next, according to Kotkova Striteska and Prokop [52], the effectiveness of firms’ science and research activities depends on their employees, their potential, and their ability to learn. Therefore, it is crucial for firms in Central and Eastern Europe to invest in the quality of workers and develop their potential. Increased employees’ skills and potential would subsequently help firms to better absorb external knowledge and technologies. To spur external absorptive capacity, it is also vital for firms to focus on the following activities [90, 92]: a) identifying and recognizing value of externally generated knowledge (e.g. through probing, informal interactions with industry actors, studying patent literature); b) learning from and with external partners including for example suppliers, customers, and competitors (e.g. through cooperation, co-invention, and networking); c) transferring knowledge back to the organization (e.g. through pacing the partners).

We also propose firms focus on the proper definition of formalized (written) business strategies with clear key performance indicators. It could increase firms’ ability to exploit more various internal and external sources, including internal and external R&D expenditures, acquisition of fixed and intangible assets and employee’s trainings. In this case, it is necessary to focus on firms´ transformational leaders (such as CEOs, executives and their top management teams—TMT) that are crucial in business operations, e.g. because they support the innovation teams by involving resources in social relationships between firm members [93]. According to Kang et al. [94], TMT play a key role in firms´ strategic decisions and corresponding outcomes. It is due to the fact that firms´ transformational leaders have the ultimate responsibility to set strategic directions, make strategic decisions, and create organizational cultures that foster or inhibit innovation.

For firms to gain access to additional knowledge resources and to benefit, for example, from knowledge spill overs, cost sharing or from the goodwill of their partners, we recommend firms to participate in firms´ groups, which were revealed as important for firms in CEE countries. Moreover, firms that are part of a larger firm group are better informed on the capabilities of potential partners (e.g., through knowledge pooling) and the activities of other members of their group. It could subsequently increase their innovation capacity [95]. Regarding the creation of external linkages, we also propose firms´ to build not only internal social capital but also external social capital which is essential for accessing external resources and overcoming uncertainty. Moreover, external social capital can yield access to important resources, opportunities, ideas, trends, and information [96, 97].

## 6 Conclusions

In this article, we expanded state of the art in the areas of open innovation and firms´ absorptive capacity in the case of selected catching-up CEE countries by using new proposed two-staged approach combining artificial neural networks and random forests. It helped us to answer the question whether firms´ from catching-up CEE countries still rely more on internal sources or whether the importance of external sources has increased and the external absorption capacity of firms has been strengthened. Despite there is a growing consensus among researchers that external knowledge sources such as external R&D and (foreign) technologies play a key role in enhancing firms´ innovation, we showed that firms´ from CEE countries still differ. Conversely, we confirmed the assumption that internal research and development overcome external resources in its importance.

Above findings show us the necessary paths for future research, which should focus primarily on the reasons why internal resources are still so important for firms from Central and Eastern Europe and why they are closed to external resources. It is clear that social capital has not yet been deepened in these countries, primarily among external partners. Therefore, we recommend that future research also focus on building external social capital and external absorption capacity in these countries. In this way, the role of firms´ transformational leaders should be also taken into account. When considering the current pandemic situation, future research should also consider alternative ways of communication and collaboration, where online meetings are growing in importance. Moreover, to verify the proposed methodological approach, future research could also contain similar analyses across selected European (primarily Western European) countries. In addition, the proposed combination of methods should be verified on the other data sources as well.

This study also contains some limitations. The lower overall correct predictions percentage of training and testing data is first limitation of our study. Second, this research did not consider the tuning of the structure and parameters of a neural network and random forests. These are, for example, cross-validation [98, 99], bootstrapping [100, 101]; or improved genetic algorithms [102, 103]. For these reasons, we acknowledge that it may primarily be a preliminary study providing novel approach for measuring firms´ innovation performance, which could be significantly advanced in the future research. Third limitation may be that we did not include more control variables that would take into account other factors of the innovation process. Fourth limitation is that we did not distinguish between selected countries and analysed aggregated datasets.
